# Evaluation of aflibercept treatment outcomes using optical coherence tomography angiography in macular telangiectasia type 1

**DOI:** 10.1186/s12886-026-04634-x

**Published:** 2026-01-23

**Authors:** Tuğba Aydoğan Gezginaslan, Yunus Emre Tüzün, Erdinç Bozkurt, Utku Limon, Betül İlkay Sezgin Akçay

**Affiliations:** 1 University of Health Sciences, Umraniye Training and Research Hospital, Istanbul, Turkey; 2 Dünyagöz Hospital, Konya, Turkey

**Keywords:** Aflibercept, Macular telangiectasia, Macular vessel density, Optical coherence tomography angiography

## Abstract

**Purpose:**

To evaluate the anatomical, functional, and vascular effects of intravitreal aflibercept in macular telangiectasia (MacTel) type 1 using optical coherence tomography angiography (OCTA).

**Methods:**

This retrospective study included nine eyes of nine patients with MacTel type 1 who underwent a single intravitreal aflibercept injection. Best-corrected visual acuity (BCVA), central macular thickness (CMT), and macular vessel density in superficial and deep capillary plexuses (SVD and DVD, respectively) were measured before and one month after treatment using swept-source OCT and OCTA.

**Results:**

Mean BCVA improved from 0.4 ± 0.3 to 0.2 ± 0.2 logMAR (*p* = 0.003) and CMT decreased from 371.4 ± 112 to 237.9 ± 55.1 μm (*p* = 0.003). Temporal SVD (*p* = 0.021) and temporal DVD (*p* = 0.020) showed statistically significant increase. BCVA change showed a strong positive correlation with CMT change (ρ = 0.70, *p* = 0.034). A moderate negative correlation between the change in BCVA (logMAR) and the change in inferior DVD did not achieve statistical significance (ρ= -0.635, *p* = 0.066).

**Conclusion:**

A single intravitreal aflibercept injection led to significant anatomical and functional improvements in MacTel type 1, with a marked increase in vessel density in the temporal quadrant. This study indicates that the characteristics of retinal microvasculature may be modified following aflibercept injection in MacTel type 1 eyes, as observed through OCTA.

## Introduction

Macular telangiectasia (MacTel) type 1 is predominantly a unilateral condition with a higher incidence in males. It is characterized by perifoveal focal capillary dilation and microaneurysms, which subsequently lead to cystoid macular edema (CME). Additionally, patchy capillary ischemia and lipid exudation may be observed in conjunction with CME. Both the superficial and deep retinal capillary circulations can be affected [1–3]. 

Currently, there is no proven method in the treatment of MacTel type 1. Various anti-vascular endothelial growth factor (anti-VEGF) agents, including bevacizumab,[4–8] ranibizumab, [9, 10] and aflibercept,^11–13^ have been employed in the management of CME associated with MacTel type 1. While these agents have generally demonstrated efficacy, aflibercept has shown particular effectiveness in cases that are refractory to bevacizumab or ranibizumab [11–13]. Aflibercept blocks both VEGF and placental growth factor (PIGF), in contrast to bevacizumab and ranibizumab, which are restricted to inhibiting only the VEGF-A isoform.

Optical Coherence Tomography Angiography (OCTA) is a non-invasive technique employed to examine the vascular structures of the macula. This method facilitates the visualization of both the superficial and deep capillary plexuses. The application of OCTA in conditions such as MacTel offers significant advantages in diagnosis due to its repeatability, non-invasive nature, and ease of execution [6, 10, 11, 14–17]. Ultimately, OCTA may prove beneficial in assessing the efficacy of treatment in diseases affecting macular capillary plexus.

This study is the first to specifically assess the changes in the macular capillary plexus following aflibercept injection using OCTA in MacTel type 1.

## Methods

A cohort of nine consecutive patients diagnosed with MacTel type 1 between 2010 and 2025 was included in the study. The study was conducted in accordance with the Declaration of Helsinki and was approved by the local ethics committee (no. B.10.1.TKH.4.34.H.GP.0.01/178) on June 24, 2025. Informed written consent was obtained from all participants.

All patients underwent a comprehensive ophthalmologic evaluation, which included measurements of best corrected visual acuity (BCVA) with an Early Treatment Diabetic Retinopathy Study chart and intraocular pressure (IOP), a complete biomicroscopic examination, color fundus photography, fluorescein angiography (FA), optical coherence tomography (OCT), and OCTA.

Eyes with preexisting ocular conditions, including degenerative macular diseases, uveitis, glaucoma, degenerative myopia, diabetic retinopathy, hypertensive retinopathy, and other retinal vascular diseases, as well as those with a history of ocular trauma, surgery or retinal laser treatment, were excluded from the study. All patients had a history of receiving injections; however, individuals who had been administered any type of intravitreal injections in the six months leading up to the study were excluded from participation.

OCT and OCTA measurements were acquired utilizing swept-source OCT (DRI Triton, Topcon, Japan). An internal fixation target was employed to enhance reproducibility. The quality of the scans was evaluated, and any image with a quality value below 40 was excluded from the study.

OCTA scans were acquired from a 6.0 × 6.0 mm cube centered on the fovea. The IMAGEnet 6 software, version 1.02.2, automatically generated en-face images of the retinal vasculature from both the superficial and deep retinal layers to delineate the superficial capillary plexus and the deep capillary plexus. Superficial vessel density (SVD) was measured from 2.6 μm beneath the internal limiting membrane to 15.6 μm beneath the interface of the inner plexiform layer (IPL) and inner nuclear layer (INL). Deep vessel density (DVD) was measured from 15.6 μm beneath the IPL/INL to 70.2 μm beneath the IPL/INL. The software provided SVD and DVD measurements for the foveal region (central, 1 mm diameter) and the parafoveal regions (3 mm diameter), categorized into superior, inferior, temporal, and nasal quadrants. Vessel density (VD) was calculated as the percentage of the area occupied by the vessels.

Segmentation errors during OCTA analysis were manually corrected and verified by the same experienced grader to ensure accurate retinal layer and vascular delineation, minimizing artifacts affecting vessel density measurements.

The criteria for administering intravitreal aflibercept (Eylea 2 mg; Bayer, Leverkusen, Germany) injection included the presence of visible leakage on FA that corresponded to CME observed in OCT. One-month post-injection, all ophthalmologic evaluations and OCT/OCTA were repeated, except for FA. Changes in BCVA, CMT and OCTA parameters were evaluated before and after the treatment.

### Statistical analysis

Categorical variables were presented as numbers and percentages, while numerical variables were expressed as mean ± standard deviation (range). The normality of data was assessed using the Shapiro-Wilk test. The Wilcoxon signed-rank test was used to compare BCVA, CMT, SVD and DVD values before and after the intravitreal injection. Correlations between variables were determined using Spearman’s correlation coefficient. A p-value of < 0.05 was considered statistically significant.

## Results

**Fig. 1:**
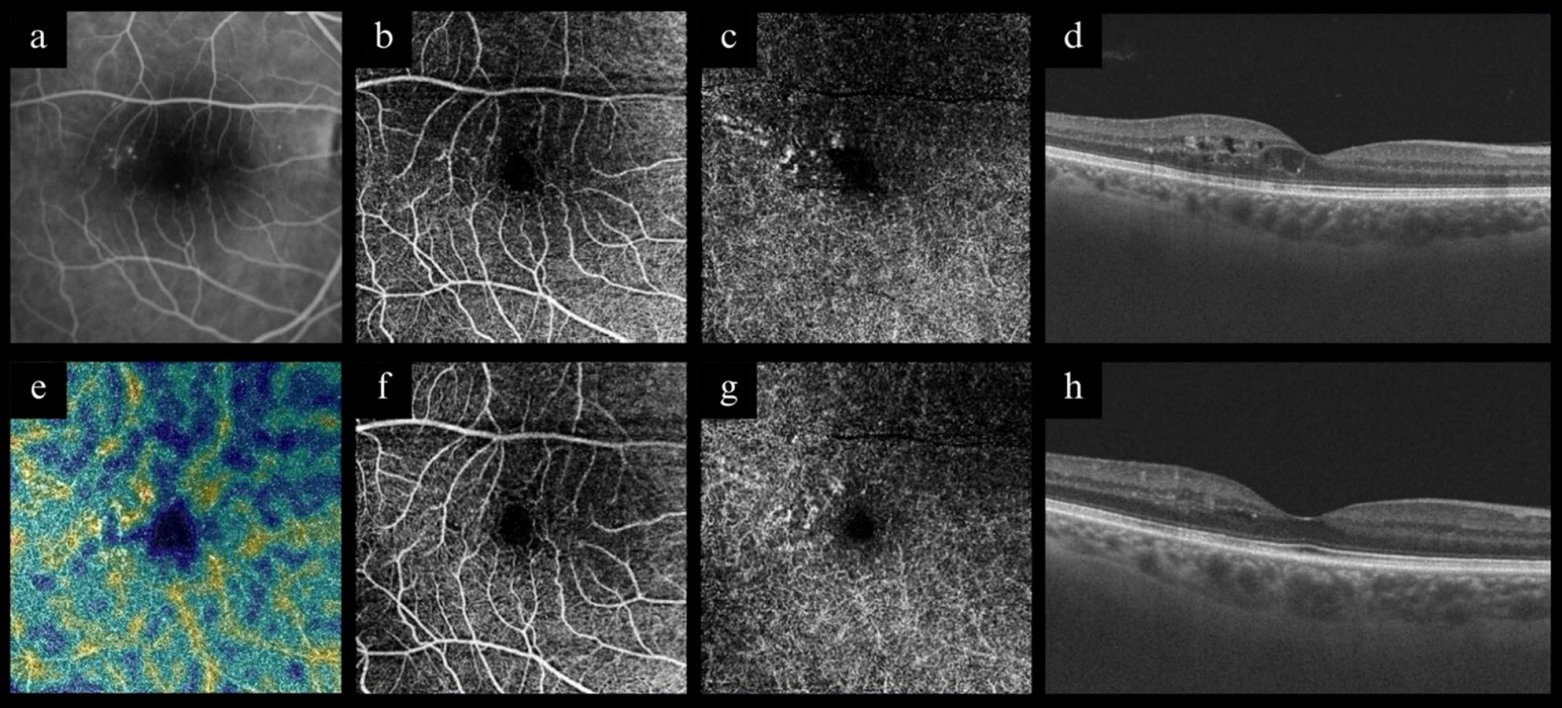
Effect of aflibercept on the right eye of the Patient 1. (**a**) Fluorescein angiography frame at 43 s post-dye injection showing leaky microaneurysms. Optical coherence tomography angiography (OCTA) showing microaneurysms, rarefied capillary plexus, and enlarged capillary-free zone in the superficial capillary plexus (**b**) and deep capillary plexus (**c**) prior to treatment. (**d**) Optical coherence tomography (OCT) B-scan showing intraretinal cystoid edema before treatment. (**e**) OCTA density map before treatment. OCTA showing reduction in microaneurysms, increase in vessel density, and more defined and rounded capillary-free zone in the superficial capillary plexus (**f**) and deep capillary plexus (**g**) after treatment. (**h**) OCT B-scan showing decrease in intraretinal cystoid edema after treatment

Nine eyes of 9 patients were included in this study. The demographic and baseline clinical characteristics of the study population are presented in Table 1. Pre- and post-injection BCVA, CMT and macular vessel density values are shown in Table 2. Mean visual acuity declined from 0.4 ± 0.3 to 0.2 ± 0.2 logMAR (*p* = 0.003), while CMT decreased from 371.4 ± 112 to 237.9 ± 55.1 μm (*p* = 0.003). Only the changes in temporal SVD and DVD reached to statistical significance (*p* = 0.021 for SVD and *p* = 0.02 for DVD). The findings from a representative case are presented in Fig. 1.

**Table 1:** Demographics and clinical characteristics of the patients

	Mean ± SD (range)
Age, years	65 ± 10 (47–75)
Male/Female, n	4/5
Baseline BCVA, logMAR	0.4 ± 0.3 (0.1-1.0)
Baseline CMT, µm	371.4 ± 112 (250.6-567.4)

SD, standard deviation; n, number; BCVA, best corrected visual acuity; logMAR, logarithm of minimum angle of resolution; CMT, central macular thickness

**Table 2:** Changes in Best-corrected visual acuity, central macular thickness and macular vessel density

Mean ± SD	Pre-injection	Post-injection	*p*
BCVA, logMAR	0.4 ± 0.3	0.2 ± 0.2	0.003*
CMT, µm	371.4 ± 112	237.9 ± 55.1	0.003*
SVD, %			
Central	15.69 ± 4.87	16.98 ± 3.53	0,260
Superior	37.18 ± 3.88	38.45 ± 4.06	0.260
Temporal	37.12 ± 7.23	42.40 ± 2.20	0.021*
Inferior	38.67 ± 6.34	40.33 ± 2.22	0.594
Nasal	36.06 ± 5.35	39.43 ± 3.66	0.086
DVD, %			
Central	15.45 ± 5.62	17.05 ± 4.62	0.441
Superior	34.25 ± 6.63	37.52 ± 6.72	0.214
Temporal	33.33 ± 7.00	40.46 ± 1.48	0.020*
Inferior	34.13 ± 8.56	37.62 ± 4.84	0.314
Nasal	35.64 ± 8.05	39.01 ± 4.94	0.110

SD, standard deviation; BCVA, best-corrected visual acuity; CMT, central macular thickness; logMAR, logarithm of minimum angle of resolution; SVD, superficial vessel density; DVD, deep vessel density.

*p < 0.05

The change in BCVA (logMAR) demonstrated a strong positive correlation with the change in CMT (ρ = 0.70, *p* = 0.034). No statistically significant correlation was found between the change in BCVA (logMAR) and the changes in SVD and DVD values. A moderate negative correlation between the change in BCVA (logMAR) and the change in inferior DVD did not achieve statistical significance (ρ= -0.635, *p* = 0.066) (Table 3). Similarly, no statistically significant correlation was detected between the change in CMT and the changes in SVD and DVD values. A moderate negative correlation between the change in CMT and the change in inferior DVD also did not reach statistical significance (ρ= -0.62, *p* = 0.077) (Table 4).

**Table 3:** Correlations between the changes in Best-Corrected visual acuity and central macular thickness and macular vessel density

BCVA, logMAR	ρ	*p*
CMT, µm	0.70	0.034*
SVD, %		
Central	-0.017	0.965
Superior	-0.043	0.913
Temporal	0.318	0.405
Inferior	-0.129	0.741
Nasal	0.163	0.675
DVD, %		
Central	0.266	0.489
Superior	0.344	0.365
Temporal	0.077	0.843
Inferior	-0.635	0.066
Nasal	0.283	0.460

BCVA, best-corrected visual acuity; logMAR, logarithm of minimum angle of resolution; CMT, central macular thickness; SVD, superficial vessel density; DVD, deep vessel density.

*p < 0.05

**Table 4:** Correlations between the changes in central macular thickness and Best-Corrected visual acuity and macular vessel density

CMT, µm	ρ	*p*
BCVA, logMAR	0.70	0.034*
SVD, %		
Central	0.15	0.700
Superior	-0.08	0.831
Temporal	-0.07	0.865
Inferior	-0.18	0.637
Nasal	-0.20	0.606
DVD, %		
Central	0.03	0.932
Superior	0.48	0.187
Temporal	-0.25	0.516
Inferior	-0.62	0.077
Nasal	0.00	1.000

CMT, central macular thickness; BCVA, best-corrected visual acuity; logMAR, logarithm of minimum angle of resolution; SVD, superficial vessel density; DVD, deep vessel density.

*p < 0.05

## Discussion

To the best of our knowledge, this is the first study to evaluate the effect of aflibercept on macular vessel density in patients with MacTel type 1. In a previous study, Kowalczuk et al. investigated the effects of aflibercept and characterized the ocular angiogenic profile of eyes affected by MacTel type 1. They assessed baseline OCTA measurements in patients with MacTel type 1; however, they did not provide quantitative OCTA-based vessel density measurements following the administration of aflibercept [11]. Our results indicated that a single dose of aflibercept injection not only led to functional and anatomical improvements but also resulted in an increase in vessel density.

Matet et al. were the first to report microvascular changes in the macula of eyes affected by MacTel type 1 using OCTA. Like our cohort of patients, Matet et al. identified telangiectasias within the deep capillary plexus, as well as a rarefied capillary plexus and enlarged capillary-free zones in both the superficial and deep capillary plexuses [16]. Furthermore, some studies have demonstrated that the macular vessel density, in both superficial and deep layers, is reduced compared to that in healthy eyes [14, 17]. 

In this study, patients with MacTel type 1 were administered a single dose of intravitreal aflibercept, and macular vascular changes were assessed using OCTA. As a result, both anatomical and functional improvements were observed, including enhanced visual acuity and a reduction in CMT. Few studies assessing the impact of aflibercept on MacTel type 1 eyes have demonstrated similar improvements, with some cases being resistant to other anti-VEGF treatments [11–13]. On the other hand, studies on bevacizumab have yielded inconsistent findings. While some studies have indicated that bevacizumab is advantageous, [4, 6] others have found no notable effects [5, 7, 8]. However, aflibercept was included in just two case reports and one case series [11–13]. Although it proved effective in refractory cases, it cannot be asserted that aflibercept is superior. Research on the application of ranibizumab in individuals with MacTel type 1 is scarce, and the results from these studies show variability [9, 10]. 

The most important finding in our study was the increase in temporal macular vessel density following aflibercept injection. Although MacTel type 1 initially impacts the deep capillary plexus, both the superficial and deep capillary plexuses exhibited improvement. Nonetheless, a statistically significant enhancement was observed only in the temporal quadrant. Gass et al. previously noted that MacTel type 1 disease tends to originate in the temporal quadrant [2]. Therefore, the statistically significant enhancement observed in the temporal quadrants might be attributed to their greater involvement. Previous studies involving aflibercept or other anti-VEGF agents did not evaluate vessel density post-treatment [11–13]. 

A moderate correlation was observed between the changes in visual acuity and macular thickness (ρ = 0.7, *p* = 0.034). In contrast, the relationship between the changes in visual acuity and macular vessel density parameters did not reach statistical significance. These results imply that short-term visual acuity improvements are more directly attributed to macular oedema resolution than macular vessel density changes. Matet et al. discovered significant correlations between visual acuity and both superficial and deep macular vessel densities [16]. Similarly, Guo et al. found significant correlations between visual acuity and CMT, SVD, DVD, and other OCT parameters. However, these studies were cross-sectional and did not explore the correlations between changes in these parameters, especially after a treatment [17]. 

The limitations of this study include the small sample size, attributable to the rarity of the condition, and the administration of only a single aflibercept injection. The limited number of injections—only two patients received a second dose—restricted the ability to evaluate longer-term effects. Another limitation lies in interpreting the observed post-injection increase in macular vessel density, as it is difficult to determine whether this reflects a true vascular improvement or is partly due to enhanced visualization of microvascular structures following the resolution of CME-related artifacts, such as masking or shadowing. This potential confounding factor should be considered when analyzing vessel density measurements obtained using OCTA. In conclusion, more extensive longitudinal studies with larger sample sizes and multiple injections are needed to better understand the dynamic relationships among macular thickness, vessel density, and visual acuity.

## Conclusions

Aflibercept improved visual acuity and reduced macular thickness in MacTel type 1, but the most notable finding was the increase in macular vessel density—especially in the temporal quadrant, the region most affected by the disease. These results suggest that aflibercept may enhance macular perfusion in addition to resolving edema. Further studies are required to determine the clinical significance of these vascular changes and the utility of OCTA in treatment monitoring.

## Data Availability

The data that support the findings of this study are available from the corresponding author, AT, upon reasonable request.

## Abbreviations

BCVA: Best–Corrected Visual Acuity
CMT: Central Macular Thickness
CME: Cystoid Macular Edema
DVD: Deep Vessel Density
FA: Fluorescein Angiography
ILM: Internal Limiting Membrane
INL: Inner Nuclear Layer
IOP: Intraocular Pressure
IPL: Inner Plexiform Layer
MacTel: Macular Telangiectasia
OCT: Optical Coherence Tomography
OCTA: Optical Coherence Tomography Angiography
PIGF: Placental Growth Factor
SD: Standard Deviation
SVD: Superficial Vessel Density
VEGF: Vascular Endothelial Growth Factor
VD: Vessel Density
